# Glymphatic system dysfunction associated with cognitive impairment in chronic tinnitus patients

**DOI:** 10.3389/fnins.2024.1455294

**Published:** 2024-09-06

**Authors:** Yinjuan Du, Zhichun Huang, Yuanqing Wu, Yuan Xue, Zigang Che

**Affiliations:** ^1^Department of Otolaryngology-Head and Neck Surgery, Zhongda Hospital, Southeast University, Nanjing, China; ^2^Department of Otolaryngology, Nanjing First Hospital, Nanjing Medical University, Nanjing, China; ^3^Department of Otolaryngology, Nanjing Pukou People’s Hospital, Nanjing, China; ^4^Department of Radiology, Nanjing Tongren Hospital, School of Medicine, Southeast University, Nanjing, China

**Keywords:** chronic tinnitus, magnetic resonance imaging, diffusion tensor imaging, glymphatic system, cognitive impairment

## Abstract

**Background:**

The glymphatic system has been regarded as a pivotal factor in the pathogenesis of neurodegenerative diseases. Given the heightened risk of cognitive impairment in chronic tinnitus patients, the possible alterations of the glymphatic system in tinnitus patients remain elusive. This study was designed to evaluate glymphatic dysfunction in chronic tinnitus patients using the diffusion tensor imaging (DTI) along the perivascular space (DTI-ALPS) approach.

**Methods:**

Fifty chronic tinnitus patients and 50 age, sex, and education-matched healthy controls (HCs) with normal hearing thresholds were recruited. The DTI-ALPS was calculated from each group. We investigated the differences in the DTI-ALPS index between the tinnitus patients and HCs. The relationships between the DTI-ALPS index and specific cognitive performance were further assessed.

**Results:**

There were significant differences in the DTI-ALPS index between the two groups. The DTI-ALPS index was significantly lower in the tinnitus group than in HCs group (*p* < 0.01). In addition, the Dyyproj index was significantly higher in the tinnitus group than in the HC group (*p* < 0.01). In chronic tinnitus patients, the decreased DTI-ALPS index was negatively associated with worse TMT-B scores (*r* = −0.309, *p* = 0.039). Moreover, the increased Dyyproj index was negatively correlated with the reduced AVLT performances (*r* = −0.413, *p* = 0.005).

**Conclusion:**

In this current study, glymphatic system activity in chronic tinnitus was investigated for the first time using DTI-ALPS index. Significant decrease in glymphatic system function was detected in chronic tinnitus, which correlated well with the specific cognitive performance. The current study may provide pivotal imaging markers for chronic tinnitus with cognitive impairment.

## 1 Introduction

Chronic tinnitus refers to the phantom ringing, buzzing or hissing sensation in the absence of external source (Møller, 2007; Wegger et al., 2017; Bauer, 2018). Tinnitus patients often suffer from depression, anxiety, and sleep problem that significantly impair the quality of daily life (Reynolds et al., 2004; Langguth, 2011; Langguth et al., 2013; Zeman et al., 2014; Bhatt et al., 2017). Previous research has identified tinnitus as influencing cognitive domains such as working memory and executive function (Hallam et al., 2004; Rossiter et al., 2006; Wang et al., 2018). That is, cognitive dysfunction of tinnitus patients could result in brain changes and added the likelihood of developing dementia or other neurodegenerative diseases (Jafari et al., 2019; Malesci et al., 2021). However, the neurophysiological mechanism of cognitive impairment associated with tinnitus remains unknown.

Recent studies have shown that glymphatic system plays a pivotal role in removing metabolic waste from the brain (Rasmussen et al., 2018). The glymphatic system involves the interaction between cerebrospinal fluid (CSF) and brain interstitial fluid (ISF). The fluid then exits the brain parenchyma through venous perivascular spaces, clearing waste such as amyloid beta (Aβ) and tau protein into meningeal lymphatic vessels (Bakker et al., 2016). When the clearance function for Aβ and tau protein is compromised, the balance will be broken, and the accumulation and aberrant deposition of Aβ and tau will lead to a cascade of damages, ultimately resulting in cognitive decline. Dysfunction in the glymphatic system has been implicated in neurodegenerative diseases (Rasmussen et al., 2018; Ciurea et al., 2023). However, none of studies have focused on investigating brain glymphatic dysfunction in chronic tinnitus to date.

Advances in magnetic resonance imaging (MRI) have made it possible to study intrinsic brain structure and function in tinnitus. Using diffusion tensor imaging (DTI), previous studies have demonstrated white matter (WM) structural alterations in tinnitus patients (Ahmed et al., 2021). Furthermore, prior resting-state functional magnetic resonance imaging (fMRI) studies have also indicated brain reorganization at the chronic stage of tinnitus (Husain and Schmidt, 2014; Chen et al., 2016; Chen et al., 2017a; Chen et al., 2017b; Chen et al., 2018a; Lan et al., 2022a). However, none of the previous neuroimaging studies have focused on brain glymphatic function in tinnitus. Derived from MRI, DTI along the perivascular space (DTI-ALPS) offers a promising non-invasive technique for evaluating brain glymphatic function (Taoka et al., 2017), which provides an assessment of the motion of water molecules in the perivascular network. DTI-ALPS has been proposed as an effective method for reflecting the brain’s glymphatic system, which has been widely used in Alzheimer’s disease (AD) (Taoka et al., 2017), mild cognitive impairment (Liang et al., 2023), traumatic brain injury (Butler et al., 2023), and presbycusis (Xu et al., 2024). Nevertheless, the relationship between brain glymphatic system function and tinnitus using DTI-ALPS has not been investigated in previous studies.

In the current study, our objective was to use the DTI-ALPS index for evaluating glymphatic system function in chronic tinnitus patients and healthy controls (HCs) with normal hearing. According to evidences from neuroimaging and clinical research, we hypothesized that chronic tinnitus may have impaired glymphatic function linked with cognitive impairment, which could provide novel enlightenment of the neuropathological mechanisms for chronic tinnitus. The current study explored for the first time to detect glymphatic dysfunction related to cognitive impairment in chronic tinnitus using DTI-ALPS technique.

## 2 Materials and methods

### 2.1 Subjects

The current study was approved by the Ethics Committee of Nanjing First Hospital (approval number: KY20220124-05). All subjects provided written informed consent before their participation in this study. Fifty chronic tinnitus patients were included from otolaryngology department while 50 age, sex, and education well-matched HCs were recruited. All individuals were 30–70 years old, right-handed and completed more than 9 years of education. Patients had bilateral or central tinnitus without hearing loss (hearing threshold <25 dB). The hearing thresholds of both ears were assessed by puretone audiometry (PTA) at the frequencies of 250 Hz, 500 Hz, 1,000 Hz, 2,000 Hz, 4,000 Hz, and 8,000 Hz. Tinnitus severity was assessed by Tinnitus Handicap Questionnaires (THQ) (Kuk et al., 1990), which was categorized as mild, moderate or severe (McCombe et al., 2001). Twenty patients had mild tinnitus, 15 moderate tinnitus, and 15 severe tinnitus. All HCs and most tinnitus patients had normal hearing. Evaluation of tinnitus related depression and anxiety symptoms were assessed using the Self-Rating Depression Scale (SDS) and Self-Rating Anxiety Scale (SAS) (Zung, 1971; Zung, 1986).

Exclusion criteria included the following: (1) pulsatile tinnitus, hyperacusis, Meniere’s diseases; (2) ear surgery, acoustic neurinoma, use of ototoxic drugs; (3) severe smoking, alcoholism, drug addiction, stroke, head injury, AD, Parkinson’s disease (PD), epilepsy, schizophrenia; (4) other major central nervous system disorders; and (5) MRI contraindications. The demographics and clinical information of the chronic tinnitus patients and HCs are presented in Table 1.

**Table 1:** Demographics and clinical information of chronic tinnitus patients and HCs.

Items	Tinnitus (*n* = 50)	HCs (*n* = 50)	*p* value
Age (year)	50.38 ± 13.56	48.76 ± 15.21	0.575
Gender (male: female)	23/27	23/27	1
Education levels (years)	10.78 ± 1.81	10.94 ± 1.87	0.664
Tinnitus duration (months)	31.20 ± 31.00	–	–
THQ score	51.51 ± 15.64	–	–
Hearing thresholds (left)	16.60 ± 2.68	17.68 ± 3.81	0.103
Hearing thresholds (right)	16.85 ± 3.03	17.10 ± 3.48	0.703
Hearing thresholds (average)	16.73 ± 2.40	17.39 ± 3.15	0.236
Gray matter volume (% of TIV)	32.16 ± 2.05	32.75 ± 1.92	0.212
White matter volume (% of TIV)	29.58 ± 1.45	29.50 ± 1.67	0.755
Brain parenchyma volume (% of TIV)	61.73 ± 2.88	62.25 ± 3.01	0.494

Data are shown as mean ± SD. HCs, healthy controls; THQ, Tinnitus Handicap Questionnaires.

### 2.2 Cognitive assessment

The cognitive status was assessed using the Mini Mental State Exam (MMSE) (Galea and Woodward, 2005), Montreal Cognitive Assessment (MoCA) (Nasreddine et al., 2005), Auditory Verbal Learning Test (AVLT) (Schmidt, 1996), Complex Figure Test (CFT) (Shin et al., 2006), Digit Span Test (DST) (Hale et al., 2002), Trail-Making Test (TMT) A and B (Bowie and Harvey, 2006), Clock-Drawing Test (CDT) (Samton et al., 2005), Verbal Fluency Test (VFT) (Mok et al., 2004), and Digit Symbol Substitution Test (DSST) (Bettcher et al., 2011).

### 2.3 MR data acquisition

MRI data were obtained using a 3.0-T MR imaging system (Ingenia, Philips Medical Systems, Netherlands) with a 32-channel receiver array head coil. To reduce head motion and scanner noise, foam pad and earplugs were used. According to the manufacturer’s specifications, the earplugs (Hearos Ultimate Softness Series, USA) could attenuate scanner noise by almost 32 dB. The scan parameters of DTI were as follows: TR = 4,996 ms, TE = 102 ms, slices = 70, slice thickness = 2 mm, gap = 0, FA = 90°, *b*-values = 0 and 1,000 s/mm^2^, diffusion gradient directions = 32, matrix = 128 × 128, and FOV = 200 mm × 200 mm. Structural images were obtained using a high-resolution T1-weighted gradient-echo sequence and the following scan parameters: TR/TE = 9.912/4.12 ms, slices = 160, thickness = 1 mm, gap = 0, FA = 16°, matrix = 256 × 256, and FOV = 256 mm × 256 mm.

### 2.4 DTI data preprocessing

After converting the DWI-DICOM data to NIfTI format, the following preprocessing steps were carried out: noise reduction, correction for eddy current distortions, and mitigation of Gibbs artifacts. Afterwards, a brain mask was generated, removing the skull, to facilitate further data analysis. DTI parameter-maps were calculated using the FSL Diffusion Toolbox.

### 2.5 DTI-ALPS index calculation

**Figure 1:**
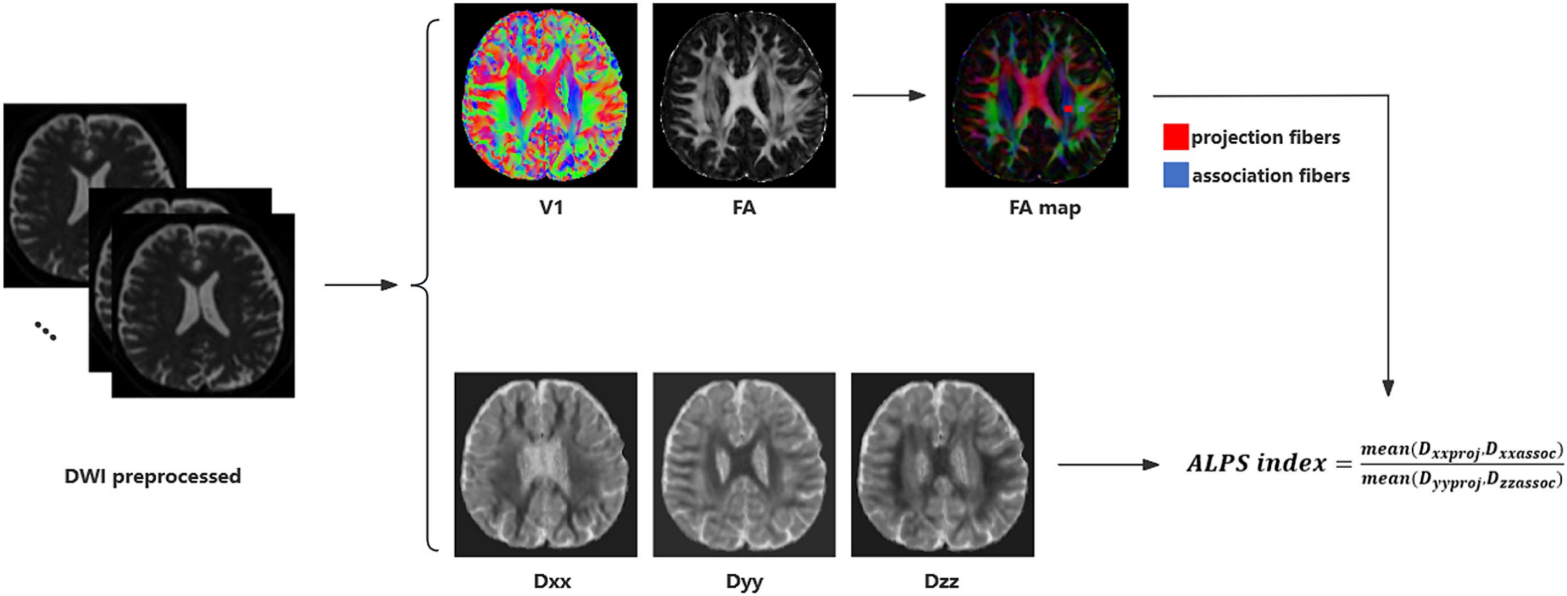
DTI-ALPS calculation method. Schematic diagram of DTI-ALPS index measurement, including DTI fitting, ROIs selection and calculation of DTI-ALPS index.

The DTI-ALPS index was calculated from diffusion-weighted imaging data using the DTIFIT tool of the FMRIB Software Library (FSL, Wellcome Centre for Integrative Neuroimaging, University of Oxford, UK).^1^ Detailed calculation process was illustrated in Figure 1 and previous study (Taoka et al., 2017; Xu et al., 2024). Initially, a rectangular region of interest (ROI) was delineated. Subsequently, the fiber orientation and diffusivities were extracted from the ROI as voxel levels along the *x*, *y*, and *z* axes. For each fiber on the same *x*-axis (projection, association, and subcortical fibers), we selected one ROI that showed the maximum orientation. All the placements of ROI were reviewed by an experienced radiologist. Diffusivity in the directions of the *x*-axis (Dxx), *y*-axis (Dyy), and *z*-axis (Dzz) of ROIs on projection fibers and association fibers were recorded as Dxxproj, Dyyproj, Dzzproj, Dxxassoc, Dyyassoc, Dzzassoc, respectively. The formula used to calculate the DTI-ALPS index is as follows:


$$\mathrm{ALPS}\;\mathrm{index}=\frac{\mathrm{mean}\left(D_{\mathrm{xxproj}},\;D_{\mathrm{xxassoc}}\right)}{\mathrm{mean}\left(D_{\mathrm{yyproj}},\;D_{\mathrm{zzassoc}}\right)}$$

### 2.6 Structural image analysis

Brain structures were calculated using VBM12 toolbox.^2^ Structural data were normalized and segmented into gray matter (GM), WM, and CSF (Ashburner and Friston, 2005). Brain parenchyma volume was measured as the sum of GM and WM volumes. The GM, WM, and brain parenchyma volume were divided by the total intracranial volume (TIV) to adjust for head size variability. T1 images were normalized to the MNI template using affine linear registration followed by Gaussian smoothing (FWHM = 8 mm).

### 2.7 Statistical analysis

The differences in demographic and clinical information were investigated using SPSS 26.0 (SPSS, Inc., Chicago, IL, USA). The chi-square test was used for categorical variables such as gender, while the independent samples t-test or Mann–Whitney U test was used for continuous variables with normally distributed data, based on normal distribution tested with the Shapiro–Wilk test. The DTI-ALPS index was calculated for each group and its correlation with specific cognitive performances was assessed using Pearson’s or Spearman’s correlation. Bonferroni correction for multiple comparisons was carried out. Statistical significance was determined using a two-tailed *p*-value of less than 0.05.

## 3 Results

### 3.1 Demographic and clinical data

The demographic and clinical data from both groups are presented in Table 1. No significant differences were detected between tinnitus patients and HCs in terms of age, gender, education level, and hearing thresholds. None of the tinnitus patients comorbid with depression or anxiety symptoms. Compared with HCs, tinnitus patients revealed significantly worse performances on the AVLT and TMT-B (*p* < 0.05) (Table 2). The other cognitive tests did not show significant decreases (*p* > 0.05).

**Table 2:** Cognitive characteristics of chronic tinnitus patients and HCs.

Items	Tinnitus (*n* = 50)	HCs (*n* = 50)	*p* value
MMSE	28.88 ± 1.27	28.82 ± 1.24	0.812
MoCA	25.74 ± 1.72	26.20 ± 1.76	0.19
AVLT	32.68 ± 7.13	35.78 ± 7.19	0.033^*^
CFT	34.40 ± 1.76	34.71 ± 1.53	0.348
CFT-delayed recall	16.83 ± 3.40	17.32 ± 3.69	0.492
DST	11.16 ± 1.60	11.80 ± 2.14	0.093
TMT-A	69.34 ± 20.44	69.44 ± 21.13	0.981
TMT-B	183.10 ± 51.57	152.36 ± 48.65	0.003^*^
CDT	3.48 ± 0.54	3.54 ± 0.54	0.582
VFT	14.39 ± 3.90	14.96 ± 3.68	0.46
DSST	69.78 ± 7.72	69.04 ± 10.16	0.683
SAS	36.52 ± 5.96	36.02 ± 6.45	0.698
SDS	38.58 ± 9.08	38.00 ± 9.14	0.751

Data are shown as mean ± SD. *p value < 0.05. HCs, healthy controls; MMSE, Mini Mental State Exam; MoCA, Montreal Cognitive Assessment; AVLT, Auditory Verbal Learning Test; CFT, Complex Figure Test; DST, Digit Span Test. TMT, Trail Making Test; CDT, Clock Drawing Test; VFT, Verbal Fluency Test; DSST, Digit Symbol Substitution Test; SAS, Self-Rating Anxiety Scale; SDS, Self-Rating Depression Scale.

### 3.2 Structural results

Compared with HCs, no significant differences of GM and WM volumes were detected in tinnitus patients (*p* > 0.05) (Table 1). After Monte Carlo simulation correction, we detected no suprathreshold voxel-wise differences of GM and WM volumes between tinnitus patients and HCs.

### 3.3 DTI analysis

**Figure 2:**
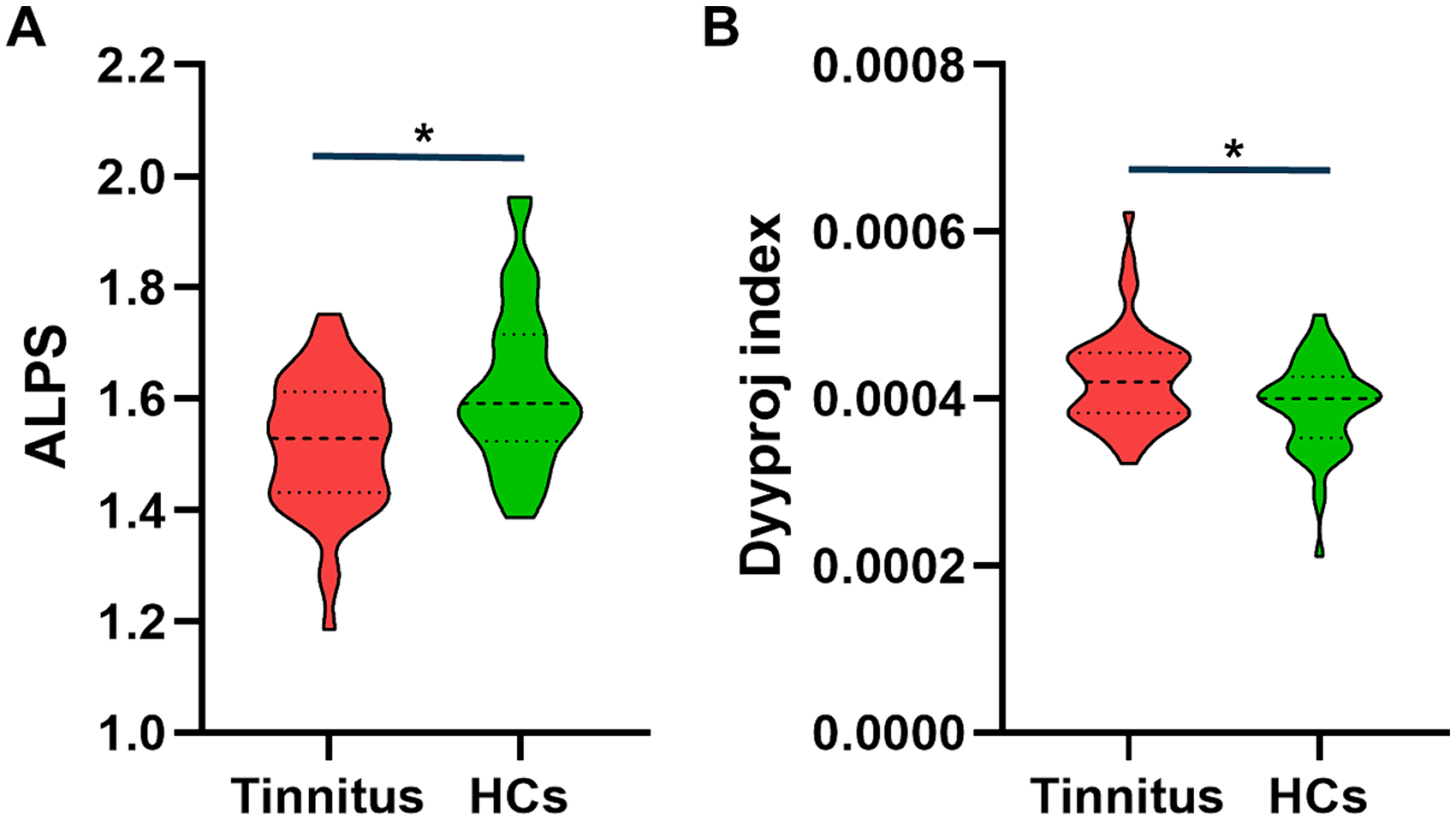
Differences between the glymphatic system functions of chronic tinnitus and HCs. **(A)** The figure shows that the DTI-ALPS index of tinnitus is significantly lower than that of HCs. **(B)** Dyyproj index was significantly higher in the tinnitus group than in the HC group.

There were significant differences in the Dyyproj and DTI-ALPS indices between two groups (Table 3). The DTI-ALPS index was significantly lower in the tinnitus group than in HCs group (*p* < 0.01) (Figure 2A). In addition, the Dyyproj index was significantly higher in the tinnitus group than in the HCs group (*p* < 0.01) (Figure 2B). No significant differences were observed in Dxxassoc, Dxxproj, Dyyassoc, Dzzassoc, or Dzzproj between the tinnitus and HC groups.

**Table 3:** Comparison of the diffusivities among tinnitus and HCs.

Diffusivity	Tinnitus (*n* = 50)	HCs (*n* = 50)	*p* value
Dxxproj	0.00060 ± 0.00005	0.00059 ± 0.00004	0.624
Dxxassoc	0.00063 ± 0.00007	0.00062 ± 0.00008	0.854
Dxxsub	0.00110 ± 0.00010	0.00108 ± 0.00013	0.895
Dyyproj	0.00042 ± 0.00006	0.00039 ± 0.00005	0.004^*^
Dyyassoc	0.00111 ± 0.00007	0.00110 ± 0.00005	0.768
Dyysub	0.00051 ± 0.00011	0.00058 ± 0.00027	0.242
Dzzproj	0.00111 ± 0.00006	0.00110 ± 0.00006	0.558
Dzzassoc	0.00038 ± 0.00007	0.00036 ± 0.00006	0.115
Dzzsub	0.00089 ± 0.00033	0.00098 ± 0.00030	0.32
ALPS index	1.51839 ± 0.11933	1.61730 ± 0.14277	0.000^*^

Data are shown as mean ± SD. ^*^p < 0.01. HCs, healthy controls; Dxxproj, diffusivity along the *x*-axis in the projection fiber; Dxxassoc, diffusivity along the *x*-axis in the association fiber; Dxxsub, diffusivity along the *x*-axis in the subcortical fiber; Dyyproj, diffusivity along the *y*-axis in the projection fiber; Dyyassoc, diffusivity along the *y*-axis in the association fiber; Dyysub, diffusivity along the *y*-axis in the subcortical fiber; Dzzproj, diffusivity along the *z*-axis in the projection fiber; Dzzassoc, diffusivity along the *z*-axis in the association fiber; Dzzsub, diffusivity along the *z*-axis in the subcortical fiber; ALPS, analysis along the perivascular space.

### 3.4 Correlation analysis

**Figure 3:**
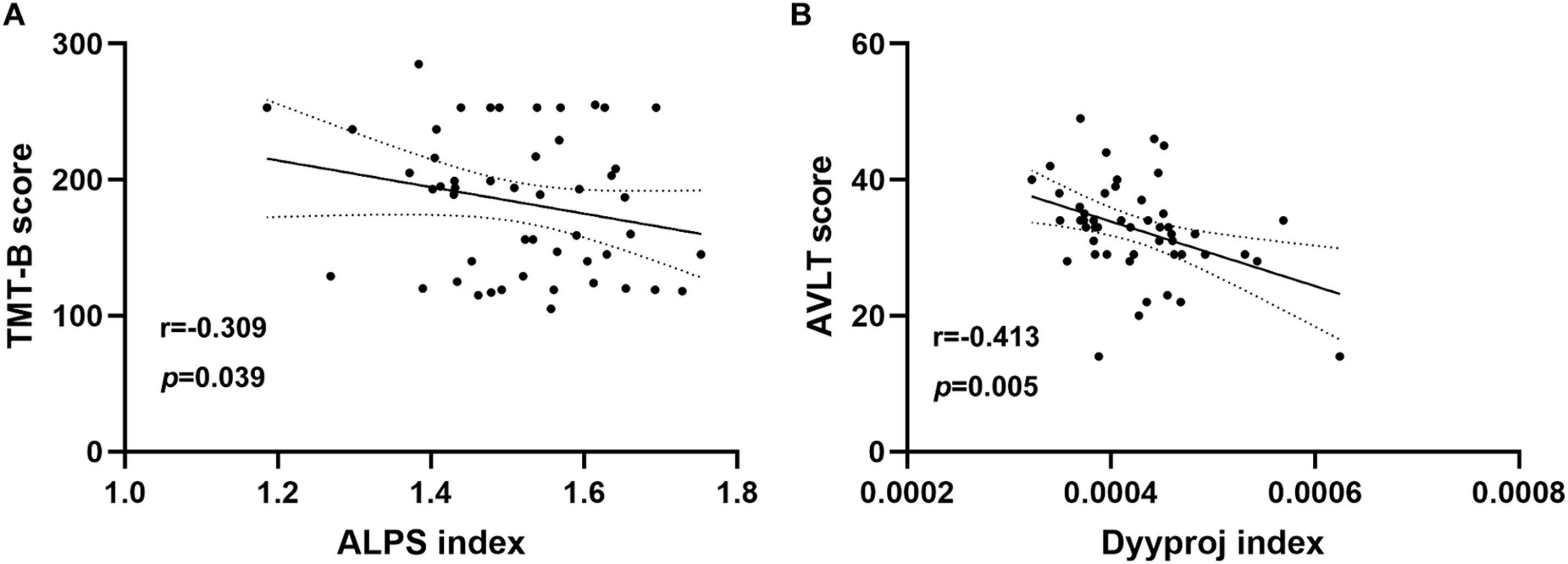
Correlation analysis. **(A)** In chronic tinnitus patients, the decreased DTI-ALPS index was negatively associated with the poorer TMT-B scores (*r* = −0.309, *p* = 0.039). **(B)** The increased Dyyproj index was negatively correlated with the reduced AVLT performances (*r* = −0.413, *p* = 0.005).

In chronic tinnitus patients, the decreased DTI-ALPS index was negatively associated with worse TMT-B scores (*r* = −0.309, *p* = 0.039) (Figure 3A). Moreover, the increased Dyyproj index was negatively correlated with the reduced AVLT performances (*r* = −0.413, *p* = 0.005) (Figure 3B). Correlation analysis was adjusted for age, gender, education, mean hearing thresholds, and GM volume. However, no other diffusivities were associated with other tinnitus characteristics or cognitive performances.

## 4 Discussion

In this study, we did not investigate any brain structural differences between tinnitus patients and HCs, which was similar with previous researches (Chen et al., 2017b; Chen et al., 2018b; Lan et al., 2022b). It could probably be due to the absence of hearing loss over the extended frequencies range and absence of hyperacusis. However, abnormal brain structural alterations due to tinnitus have also been reported in prior researches (Schmidt et al., 2018; Elmer et al., 2023). The heterogeneity of the tinnitus patients and structural MRI analytical method may result in the discrepancy. Nevertheless, the present findings suggest that glymphatic dysfunction may exist prior to brain structural changes in tinnitus without hearing loss.

The glymphatic system’s role is to clear metabolic waste and interstitial solutes from the brain parenchyma, including the CSF tau protein (Iliff et al., 2013). Increasing evidences have supported that the ALPS index is a promising alternative indicator for assessing the function of the glymphatic system and has been widely used in a variety of neurodegenerative diseases. Taoka et al. reported positive correlation between reduced DTI-ALPS index and cognitive dysfunction in AD patients for the first time (Taoka et al., 2017). Xu et al. demonstrated reduced glymphatic function in presbycusis patients with cognitive impairment compared to those with normal cognition and HCs (Xu et al., 2024). Sha et al. identified that a significant decrease in glymphatic system function was detected in children with congenital sensorineural hearing loss, which was correlated well with the age (Sha et al., 2024). Our results indicated reduced DTI-ALPS index and increased Dyyproj index in chronic tinnitus patients, which was correlated with specific neuropsychological tests (AVLT and TMT-B). Based on the aforementioned findings, DTI-ALPS is pivotal in detecting functional changes in the glymphatic system and underscoring the potential value of the ALPS index as a biological indicator of neuropathological conditions.

Multidimensional cognitive performances were conducted to evaluate the cognitive and psychological status for each subject. However, most tests showed non-significant differences between groups but the AVLT and TMT-B performances. The AVLT and TMT-B scores in tinnitus group showed significant decreases compared with the scores of the control group, suggesting that chronic tinnitus perception may cause overt impairment of memory and executive function, which was in consistent with previous studies (Chen et al., 2018b). Moreover, the correlation between AVLT, TMT-B and DTI-ALPS index had been detected, suggesting that the glymphatic system could have an impact on the memory function and information processing speed. Hsu et al. demonstrated that glymphatic system activity may act as a significant mediator in AD-related brain regions, which are responsible for memory function and information processing speed (Hsu et al., 2023). Moreover, prior studies also detected the relationships between glymphatic dysfunction and memory decline in many neurodegenerative diseases (Buccellato et al., 2022; Kamagata et al., 2022; Liang et al., 2023). Therefore, it is reasonable to speculate that chronic tinnitus with cognitive impairment may have glymphatic impairment. Nonetheless, we did not detect a direct relationship between glymphatic dysfunction and cognitive impairment in chronic tinnitus. Whether and how tinnitus plays a critical role in glymphatic function still remains to be further determined.

The present study had several limitations. First, the generalisability of the findings may be limited by the relatively small sample size. It is difficult to make causal deduction regarding the relationship between the glymphatic function and cognitive impairment in chronic tinnitus patients. Second, chronic tinnitus patients often suffer from sleep problems, which may cause glymphatic system dysfunction in prior studies (Chong et al., 2022). However, the sleep status of our tinnitus patients was not evaluated in this study. Furthermore, although this study has attempted to minimize the scanner noise with earplugs, we cannot completely prevent subjects from hearing some sound. This limitation should be taken into consideration while attempting to draw conclusions on resting-state fMRI results in auditory field. Finally, the ROI along the periventricular area was manually drawn, introducing a subjective factor to our measurement. Though the DTI-ALPS measured in the periventricular area may reflect the integrity of the focal lymphatic system, it was not possible to comprehensively assess focal perivascular spread (Taoka et al., 2024). Thus, for the evaluation of glymphatic system function, it is now considered necessary to evaluate not only the ALPS method, but also a combination of other methods such as choroid plexus volume and perivascular space volume.

## 5 Conclusion

In summary, we used the DTI-ALPS algorithm to detect a prominent decrease of the glymphatic function in chronic tinnitus patients while negative relationships between glymphatic dysfunction and specific cognitive performance were observed. DTI-ALPS index may provide useful disease progression or treatment biomarkers for tinnitus patients as an indicator of modulation of glymphatic function. Further studies are needed to focus on investigating the diagnostic and therapeutic implications of glymphatic dysfunction in tinnitus patients with cognitive impairment.

## Data Availability

The original contributions presented in the study are included in the article/supplementary material, further inquiries can be directed to the corresponding authors.

## Footnotes

^1^ https://fsl.fmrib.ox.ac.uk/fsl/fslwiki/FSL

^2^ http://www.fil.ion.ucl.ac.uk/spm
